# Divergent expression of *aristaless1* and *aristaless2* during embryonic appendage and pupal wing development in butterflies

**DOI:** 10.1186/s12915-023-01602-5

**Published:** 2023-05-11

**Authors:** Erick X. Bayala, Isabella Cisneros, Darli Massardo, Nicholas W. VanKuren, Marcus R. Kronforst

**Affiliations:** Department of Ecology & Evolution, University of Chicago, Chicago, IL 60637 USA

**Keywords:** *Heliconius*, *Aristaless*, Appendage formation, Gene duplication, Subfunctionalization, Butterfly color patterning, Wing pigmentation, Scale development

## Abstract

**Background:**

Gene duplication events are critical for the evolution of new gene functions. Aristaless is a major regulator of distinct developmental processes. It is most known for its role during appendage development across animals. However, more recently other distinct biological functions have been described for this gene and its duplicates. Butterflies and moths have two copies of *aristaless*, *aristaless1* (*al1*) and *aristaless2* (*al2*), as a result of a gene duplication event. Previous work in *Heliconius* has shown that both copies appear to have novel functions related to wing color patterning. Here we expand our knowledge of the expression profiles associated with both ancestral and novel functions of Al1 across embryogenesis and wing pigmentation. Furthermore, we characterize Al2 expression, providing a comparative framework between gene copies within the same species, allowing us to understand the origin of new functions following gene duplication.

**Results:**

Our work shows that the expression of both Al1 and Al2 is associated with the ancestral function of sensory appendage (leg, mouth, spines, and eyes) development in embryos. Interestingly, Al1 exhibits higher expression earlier in embryogenesis while the highest levels of Al2 expression are shifted to later stages of embryonic development. Furthermore, Al1 localization appears extranuclear while Al2 co-localizes tightly with nuclei earlier, and then also expands outside the nucleus later in development. Cellular expression of Al1 and Al2 in pupal wings is broadly consistent with patterns observed during embryogenesis. We also describe, for the first time, how Al1 localization appears to correlate with zones of anterior/posterior elongation of the body during embryonic growth, showcasing a possible new function related to Aristaless’ previously described role in appendage extension.

**Conclusions:**

Overall, our data suggest that while both gene copies play a role in embryogenesis and wing pigmentation, the duplicates have diverged temporally and mechanistically across those functions. Our study helps clarify principles behind sub-functionalization and gene expression evolution associated with developmental functions following gene duplication events.

**Supplementary Information:**

The online version contains supplementary material available at 10.1186/s12915-023-01602-5.

## Background

Gene duplication is a strong driving force for the evolution of new gene functions [15]. Such duplication events allow for the formation of extra copies, often alleviating the pressure imposed by the ancestral function on the single gene copy [10]. Hence, gene duplication enables interesting avenues for gene functions to evolve. The *aristaless* (*al*) gene is a major regulator of multiple developmental functions across animals. Such functions are mainly associated with appendage formation, development, and patterning of appendages [4, 7, 8, 11, 20, 22, 24, 26]. For example, velvet worms exhibit *aristaless* expression early in embryogenesis, within the cells forming leg buds. In cnidarians, the aristaless-like homeobox gene (*alx*) is involved in initiating tentacle development during embryonic growth and across adult appendage regeneration [26] highlighting the presence of this function even before the protostome/deuterostome split. Within echinoderms and vertebrates, Alx activity regulates skeletogenesis and limb patterning [11, 22]. Interestingly, several duplication events have happened across vertebrates from which novel roles, like the regulation of neurogenesis and pigmentation, have evolved for one or more copies of the gene across rodents and fishes [12, 13, 16].

Across invertebrates, Aristaless has been shown to function as a key regulator of proper patterning and appendage extension as well [4, 7, 8, 20, 24]. In flies, Aristaless was first described based on its role in the formation of a hair-like structure called the arista, which extends from the antennae [24]. Since then, *aristaless* expression in flies has been shown to be relevant for the patterning of imaginal discs (both leg and wings) and their eventual extension into future adult structures [8, 24]. Outside of flies, Aristaless has also been shown to play a role in the specification and extension of appendages. In *Gryllus*, early embryology work has shown how the expression of *aristaless* is associated with the tips of appendage buds growing out of the primary body plan [4, 19]. Similarly, research in beetles has shown that the extension and branching patterns associated with their horns are also related to Aristaless activity [20]. In summary, *aristaless* appears to be a key gene for the regulation of patterning, extension, and formation of appendages across several invertebrate species and perhaps even animals as a whole.

In Lepidoptera, the *aristaless* gene has also been duplicated [17]. This is one of the few known cases of *aristaless* duplication in invertebrates and uniquely positions butterflies to address gene function evolution following gene duplication, especially with regard to potential subfunctionalization. Previous research on *aristaless* in butterflies and moths has mainly used a non-specific antibody that targets products of both copies of the gene (in addition to other homeodomain proteins [17], masking individual functional contributions. In moths, previous work has shown that *aristaless* copies have important roles when it comes to the formation and proper patterning of the antennae [2] suggesting its relevance in appendage development. More recently, Aristaless (one or both copies of the gene) activity in butterflies has been shown to be associated with color patterning processes during wing development [17] suggesting a novel and distinct function. Furthermore, more specific approaches targeting gene transcripts have suggested that different expression patterns for the two copies of the gene, *aristaless1* (*al1*) and *aristaless2* (*al2*), are involved in patterning specific wing color elements [17, 27]. Related to this novel color patterning function, our previous work studied *al1* in detail as the key regulator of the white and yellow color pattern switch in *Heliconius* butterflies [3, 27]. As part of that work, we showed that *al1* expression and activity are needed for the proper formation of embryonic appendages in *Heliconius* [3]. This developmental characterization suggested that Al1’s novel role in color patterning is possibly achieved by altering scale maturation or elongation rates, which functionally relates to the ancestral cellular processes of appendage formation and extension. The potential connection between Al’s functions related to color pattern formation and appendage formation underscores the need for a more thorough analysis of Al1’s role in appendage formation in *Heliconius*.

Furthermore, no distinct data are available related to Al2 expression or activity in the context of appendage formation or color patterning in *Heliconius*. Given both aristaless appear to be somewhat related to color patterning, it begs the question of whether other functional aspects, like appendage development, are also shared. A proper understanding of how these genes evolved after the duplication event and/or potential regulatory or physical interactions requires that we characterize the developmental functions of both Al1 and Al2 across the distinct functions of embryonic development and color patterning. Characterizing both genes in a comparative framework between the genes, but within the same species, allows us to better understand the principles behind the developmental function of both Al1 and Al2 with respect to multiple aspects of *Heliconius* development. Furthermore, this work provides a unique evolutionary angle, providing novel insight into how gene function can change following a duplication event.

Here we characterize Al1 and Al2 expression across early embryology and within developing pupal wings of *Heliconius* butterflies. We do this by first describing the expression pattern of Al1 across multiple unexplored embryonic stages and in the wing across early pupal development where color patterning is happening. We further extend this analysis to analyze Al2 expression and protein subcellular localization in order to gain a comparative view of both copies of the gene across *Heliconius* development. Finally, analyzing the similarities and differences between Al1 and Al2 expression and subcellular localization allows us to understand the larger developmental and cellular principles behind their function in appendage formation and pigmentation.

To carry out this work, we developed a series of tools for research in *Heliconius* butterflies. We used newly developed antibodies and in situ riboprobes that specifically target both gene products to tease apart expression differences between them. Furthermore, we applied these probes and antibodies to adapted protocols that allow us to analyze multiple embryonic stages for the first time in *Heliconius* butterflies. Embryos prior to hatching were used in order to analyze expression differences between Al1 and Al2 within appendage precursors and sensory organs. We coupled our early embryology analysis with pupal wing tissue staining to provide a complete view of the differences associated with Al1 and Al2 expression and subcellular localization across development and with respect to the novel (wing coloration) and ancestral roles (appendage extension).

## Results

### Expression of Al1 and Al2 is associated with the development of sensory appendages during mid (42–48 h after deposition) embryogenesis

**Fig. 1:**
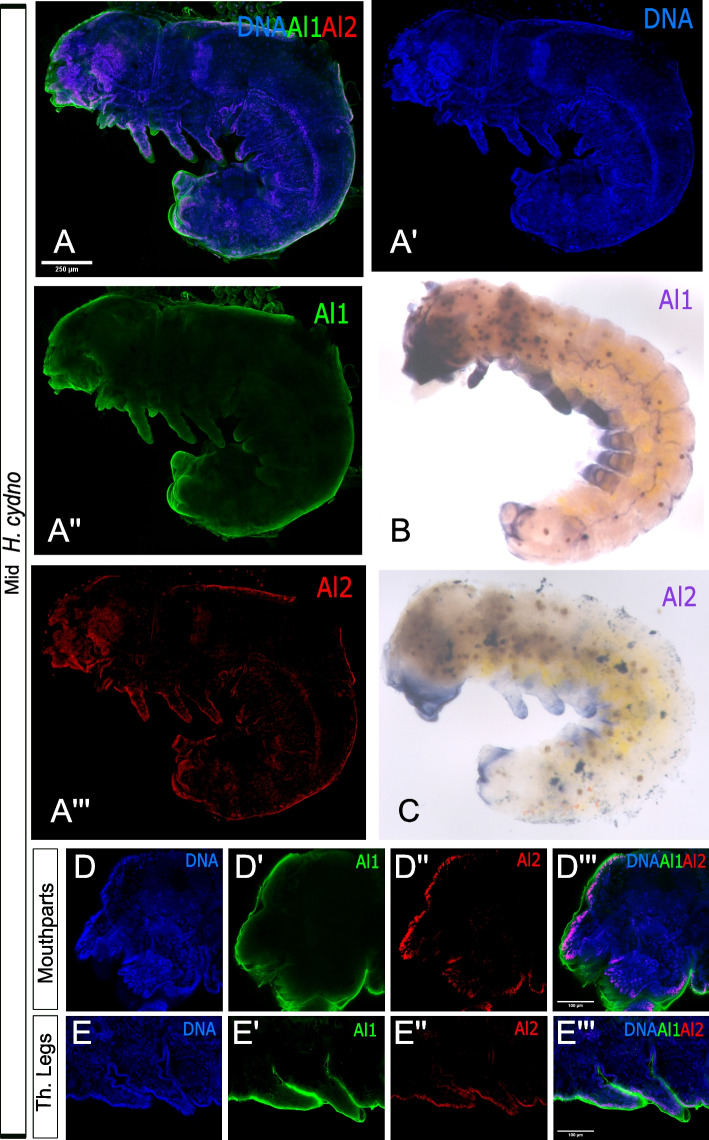
Immunodetection and in situ hybridization for Al1 and Al2 protein and transcript in mid (42–48 h after deposition) wild type *Heliconius cydno* embryos. **A** Immunodetection of Al1 and Al2 in a wild-type embryo (lateral-view). **B** Al1 in situ hybridization. **C** Al2 in situ hybridization. **D**, **E** Details on the immunodetection of Al1 and Al2 in mouthparts (**D**) and thoracic legs (**E**) are shown. Panels show detection of DNA (**A′**, **D**, **E**), Al1 (**A″**, **D′**, **E′**), Al2 (**A″′**, **D″**, **E″**), and a merge (**A**, **D″′**, **E″′**). Scale bars are shown on the merged images

Our previous antibody staining showed that Al1 expression is associated with the development of appendages across specific stages of embryogenesis [3]. We used our newly developed *al1* probe to determine whether we could recapitulate this previously observed expression pattern for Al1 [3]. Both in situs and antibody staining showed that Al1 expression was associated with sensory appendages, exhibiting higher accumulation at the distal tip (Fig. 1A″–B). This is consistent with our previous observations [3] and with the expression of *aristaless* in other insects [4, 7, 8, 20, 24]. In addition, and as previously reported for Al1 [3], the observed expression by antibody staining did not appear to co-localize with nuclei (Fig. 1A).

We analyzed the same timepoint for Al2 and observed similar expression patterns between in situ and antibody staining across the same appendages (Fig. 1A″′, C). However, different from Al1, the expression of Al2 across appendages did co-localize with nuclei. Furthermore, Al2 expression appeared weaker than Al1 when comparing both within the same individual by antibody staining (Fig. 1A) and when looking at two different embryos at the same developmental stage by in situ hybridization (Fig. 1B, C). This suggests that both Al1 and Al2 are associated with the development of sensory appendages during *Heliconius* embryogenesis. However, the differences in subcellular localization within developing appendages and detection levels between both copies (Fig. 1D, E) suggest other differences probably exist with respect to the expression pattern of both duplicates across development. To further characterize this, we analyzed earlier and later stages of embryonic development to get a clearer picture of how Al1 and Al2 expression changes across time.

### Al1 and Al2 expression patterns are temporally distinct and spatially complex but remain associated with embryonic sensory appendages across embryogenesis

**Fig. 2:**
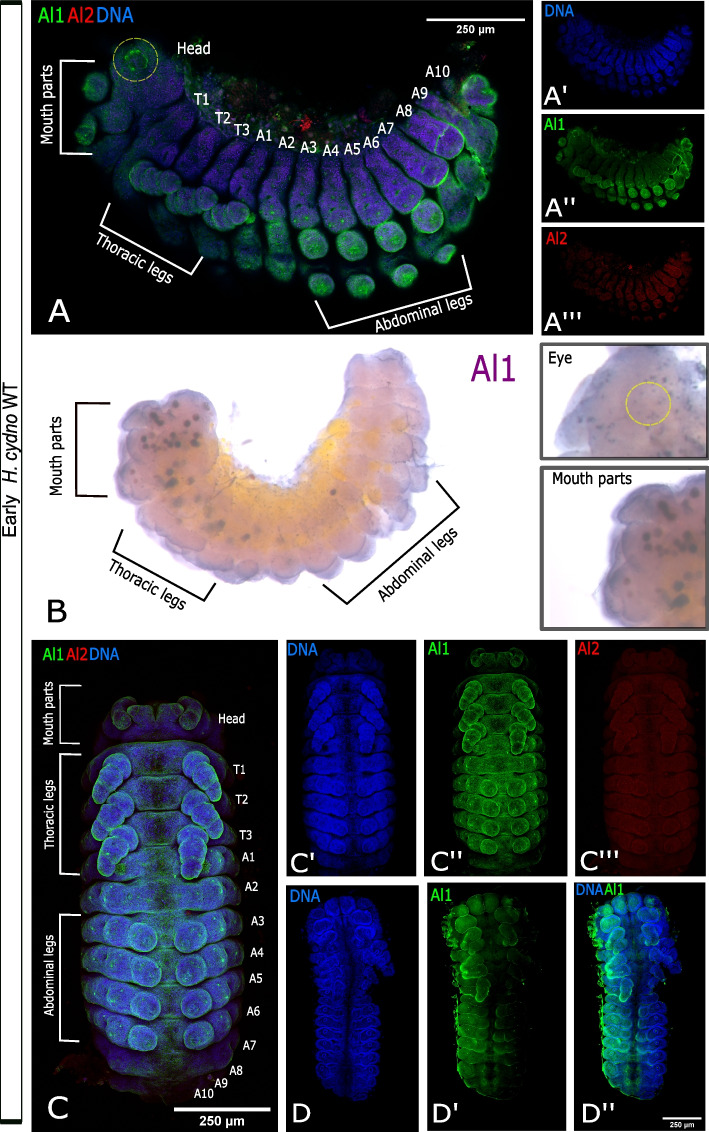
Immunodetection of Al1 and Al2 in early (36–42 h after deposition) wild type and –Al1 CRISPR *Heliconius cydno* embryos. **A** Immunodetection of Al1 and Al2 in a wild-type embryo (lateral-view). **B** Al1 in situ hybridization. **C** Immunodetection of Al1 and Al2 in a wild-type embryo (ventral view). **D** Immunodetection of Al1 in an -Al1 CRISPR embryo (data from [3]). White brackets highlight the different anatomical sections in **A** and **B**. The segments are also labeled on the merge images (Thoracic [T], Abdominal [A]). Panels show detection of DNA (**A′**, **C′**, **D**), Al1 (**A″**, **C″**, **D″**), Al2 (**A″**′,** C″′**, **D″**), and a merge (**A**, **C**, **D″**). 250-μm scale bars are shown on all merged images

We first analyzed embryos from earlier stages to determine if both Al1 and Al2 are expressed at the start of appendage growth. Our analysis of early embryos (36 to 42 h after egg deposition) first revealed that Al2 was absent or very weakly expressed across the body and appendages while Al1 was highly expressed within developing sensory appendages (Fig. 2A). Embryos at this time point showed strong expression of Al1 within multiple sensory appendages including mouth parts, thoracic legs, abdominal legs, eyes, and spines (Fig. 2A). The same pattern of expression for Al1 was observed by in situ hybridization (Fig. 2B). As previously reported, the observed expression of Al1 did not appear to co-localize with nuclei (Fig. 2A, C). Furthermore, CRISPR experiments supported the observed expression of Al1 at this time point because appendages lacking Al1 during this time point exhibited malformations and extension defects (Fig. 2D).

**Fig. 3:**
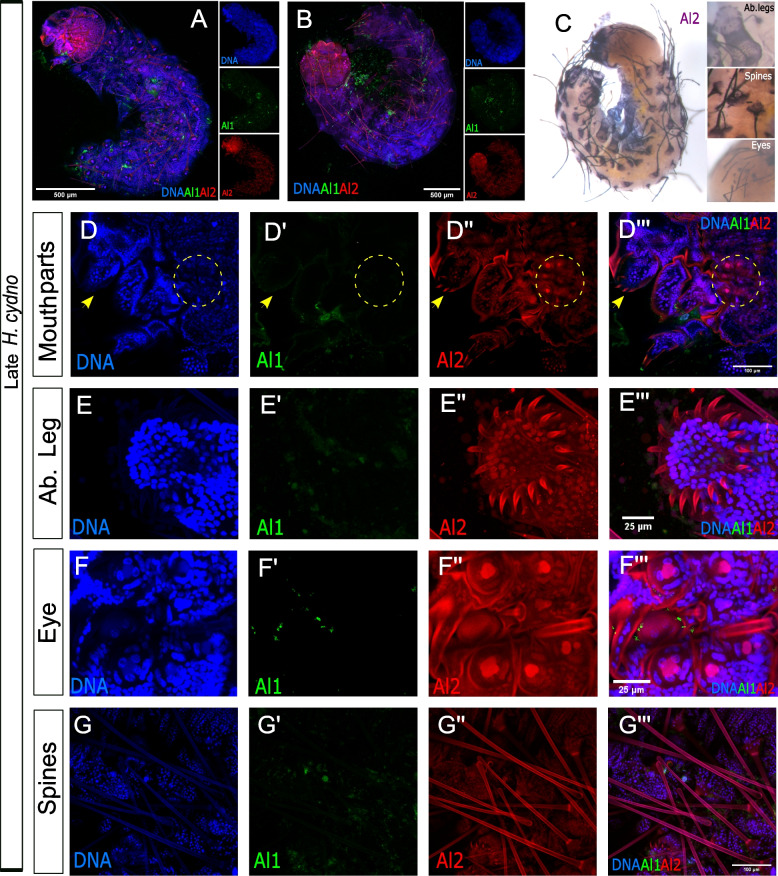
Immunodetection of Al1 and Al2 in early (48–60 h after deposition) wild-type and –Al1 CRISPR *Heliconius cydno* embryos. **A** Immunodetection of Al1 and Al2 in a wild-type embryo (lateral-view). **B** Immunodetection of Al1 and Al2 in a wild-type embryo (dorsal-view). **C** Al2 in situ hybridization of wild-type embryo (**D–G**). Immunodetection of Al1 and Al2 in specific sensory appendages (mouthparts [**D**], abdominal leg tips [**E**], eye [**F**], spines [**G**]). Panels show detection of DNA (**D**, **E**, **F**, **G**), Al1 (**C′**, **D′**, **E′**, **F′**, **G′**), Al2 (**C″**, **D″**, **E″**, **F″**, **G″**), and a merge (**A**, **B**, **C″′**, **D″′**, **E″′**, **F″′**, **G″′**). Scale bars are shown on the merged images

We also analyzed and compared Al1 and Al2 expression patterns during late-embryonic development. During late time points (48–60 h after egg deposition), we observed reduced Al1 expression across the entire embryo (Fig. 3A, B). Interestingly, during these late time points, and just a few hours before hatching, we detected strong Al2 expression both inside and outside of nuclei within multiple sensory appendages (mouth parts, thoracic legs, abdominal legs, eyes, and spines; Fig. 3A–G). The high Al2 expression across these sensory appendages was also observed by in situ hybridization (Fig. 3C). These observations across the majority of embryonic development showcase a temporal shift between the expression and possibly the activity of both copies of the gene. Such temporal shifts were also noticeable by in situ hybridization when looking across multiple stages of development using probes against both duplicates (Additional file 1: Figure S1). Furthermore, the differences we observed in terms of nuclear co-localization suggest different and dynamic spatial regulation between the duplicates.

### Al1 and Al2 exhibit unique subcellular localization tightly associated with the development of spines and eyes

Our previous work described Al1’s role in appendage extension [3]. Most of that original description focused on embryonic legs and mouthparts. Here we further examined how expression patterns of Al1 and Al2 are associated with other sensory appendages (eyes and spines) across multiple stages of embryonic development. The development of these appendages has been poorly analyzed in terms of expression in other insects. Furthermore, analyses of Al1 and Al2 expression during the development of these sensory appendages do not exist in butterflies. We coupled our co-staining approaches with high-magnification confocal microscopy to determine the expression patterns and subcellular localization of Al1 and Al2 during larval eye and spine development.

**Fig. 4:**
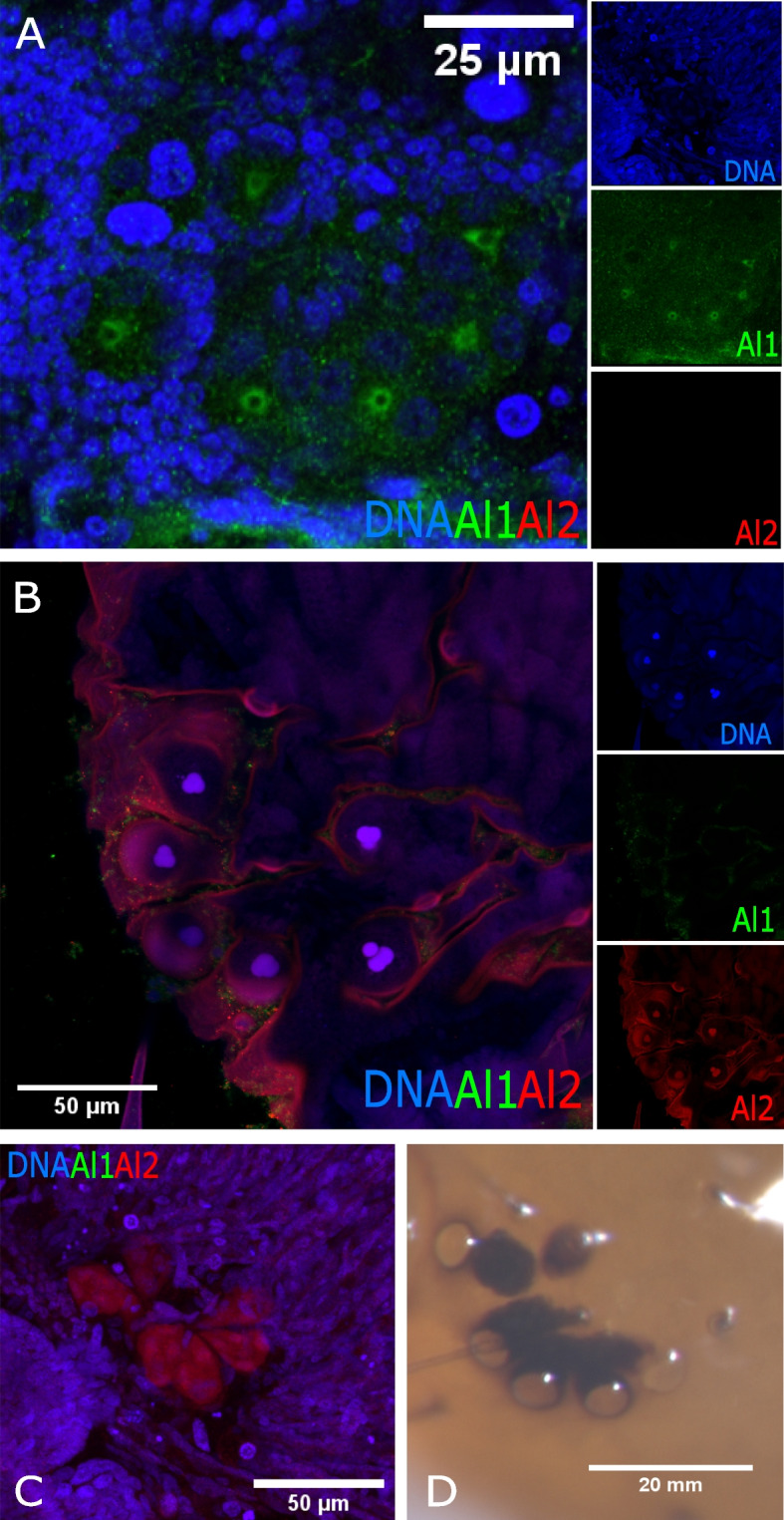
Detailed comparison of the immunodetection of Al1 and Al2 between early (36 h after deposition) and late (48 h after deposition) wild-type eyes of *Heliconius cydno* embryos. **A** Immunodetection of Al1 and Al2 in early wild-type embryonic eyes. **B** Immunodetection of Al1 and Al2 in late wild-type embryonic eyes. **C** Deeper *z*-plane view of the **B** panel showcases the detection of Al2 within the cell bodies projecting into the brain. **D** Caterpillar eye following eclosion. Panels show merged views (**A**, **B**, **C**). The insets of the merged images highlight DNA, Al1, and Al2 detection. Scale bars are shown on the merged images and Panel **D**

Similar to other appendages, eyes also exhibited strong Al1 expression early during embryonic development (Fig. 4A) and strong Al2 expression later in development (Fig. 4B). Early in eye development, Al1 was enriched in a ring-like pattern within the center of each one of the six simple eyes (ocelli) present on each side of the head. This ring-like pattern of expression was not co-localized with any of the nuclei in the center of the eye (Fig. 4A). During the late stages of embryonic development, we observed Al2 in the center of the eyes, co-localizing with the three nuclei within each eye (Fig. 4B). Furthermore, there was clear detection of Al2 within a tube under each eye going deeper into the head/brain region (Fig. 4C). These tube structures were visible within semi-transparent, freshly emerged caterpillars as a pigmented tube under the opening of the eyes (Fig. 4D).

**Fig. 5:**
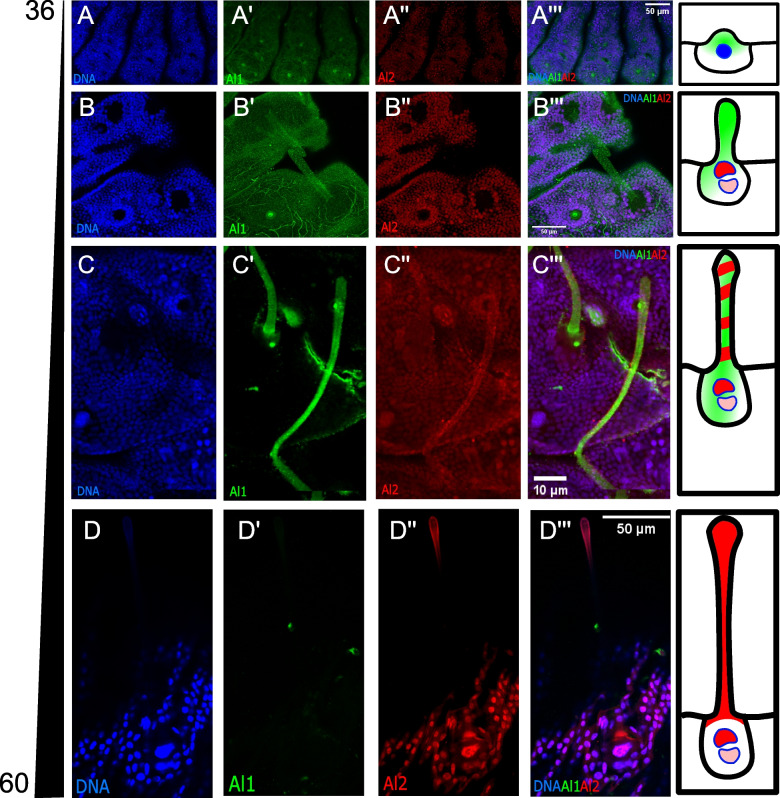
Detailed comparison of the immunodetection of Al1 and Al2 across development in wild-type spines of *Heliconius cydno* embryos. **A** Immunodetection of Al1 and Al2 in wild-type embryonic spines 36 (**A**), 48 (**B**), 50 (**C**), and 60 (**D**) h after deposition. Panels show detection of DNA (**A**, **B**, **C**, **D**), Al1 (**A′**, **B′**, **C′**, **D′**), Al2 (**A″**, **B″**, **C″**, **D″**), and a merge (**A″′**, **B″′**, **C″′**, **D″′**). Scale bars are shown on the merged images. A schematic representation of the observed subcellular detection for Al1 and Al2 is shown on the right of each time point

We also analyzed spines during their development process. Like other sensory appendages, spines also exhibited a temporal shift between strong early Al1 expression (Fig. 5A) and strong late Al2 expression (Fig. 5D). However, the bigger size of the spine cells and associated nuclei allowed us to observe several unique cellular features that we could not analyze in other appendages. As spine cells started to elongate, we observed a clear transition between Al1 and Al2 expression (Fig. 5A–D). Furthermore, it was very apparent that Al1 never co-localized with the two large nuclei that form the spine. Instead, Al1 appeared more diffuse along the entire spine body. Al2, on the other hand, was completely restricted to nuclei early in development (Fig. 5B). Then, as development continued, a shift towards extranuclear Al2 was seen while still maintaining strong nuclear co-localization (Fig. 5C, D). An intermediate state was observed as well, where both Al1 and Al2 were detected within the developing spine (Fig. 5C). Finally, during the late stages of development, one of the two nuclei that form part of the spine exhibited a higher level of Al2 than the other (the more distal nucleus within the spine exhibited higher Al2 expression; Fig. 5D) possibly suggesting differences in the regulatory role of Al2 for different cell identities involved in forming the spine.

### Al1 exhibits complex dorsal and ventral expression patterns in developing embryos

**Fig. 6:**
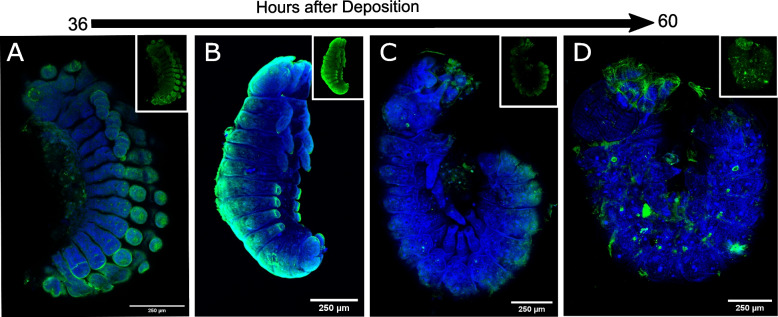
Immunodetection of Al1 across embryonic development in wild-type *Heliconius cydno* embryos highlighting detection of ventral and dorsal body sections extending within the anterior–posterior axis. Side views are shown for the immunodetection of Al1 in wild-type embryos at 36 (**A**), 42 (**B**), 50 (**C**), and 60 (**D**) hours after deposition. Panels show merged views of both DNA and Al1. Insets of just Al1 detection are also shown. Scale bars are shown on the merged images

In addition to the described patterns of localization for Al1 within appendages, we also noticed an accumulation, either ventrally or dorsally, in developing embryos that have not been described for *aristaless* in other insects. When we analyzed Al1 across embryonic development, we observed that Al1 protein expression correlated with parts of the embryos that were extending along the anterior–posterior (A/P) axis. Early in development, prior to the body fold that bends the legs inwards, we observed Al1 expression ventrally (Fig. 6A). After the inward bend, and during A/P extension, we observed an accumulation of Al1 along the dorsal side of the embryo (Fig. 6B). As A/P extension progressed, we observed a shift toward Al1 accumulation posteriorly (Fig. 6C) coinciding with the extension of the abdomen. This localization along the dorsal side faded around 60 h post-egg deposition (Fig. 6D) when the A/P extension is presumed to have concluded as the embryo is already occupying the entire physical space of the egg hours before hatching. The observed accumulation appeared more diffuse and did not coincide with nuclei, similar to what we observed in appendages.

### Al1 and Al2 exhibit a temporal shift during wing development

The observed pattern of Al2 expression, transitioning from nuclear to extranuclear, and the temporal shift between early and late embryos raised the question about whether these observations are unique to the ancestral embryonic role of *aristaless* in appendage formation. We previously described how Al1 also exhibits a novel role with respect to color patterning in pupal wings [3]. Although Al1 has been characterized developmentally as the switch between white/yellow coloration and an overall regulator of *Heliconius* wing coloration, such a novel role concerning pigmentation in *Heliconius cydno* has not been tested for Al2. However, some developmental data exist suggesting Al2 might also be involved in pigmentation [17]. Given the observed temporal transition from Al1 to Al2 in embryos, we examined whether this transition occurs in wings as well, perhaps suggesting a level of physical or regulatory interaction needed for proper function in the context of color pattern formation. This would also further provide evidence that the developmental mechanisms between ancestral and novel functions might be related, as previously suggested [3].

**Fig. 7:**
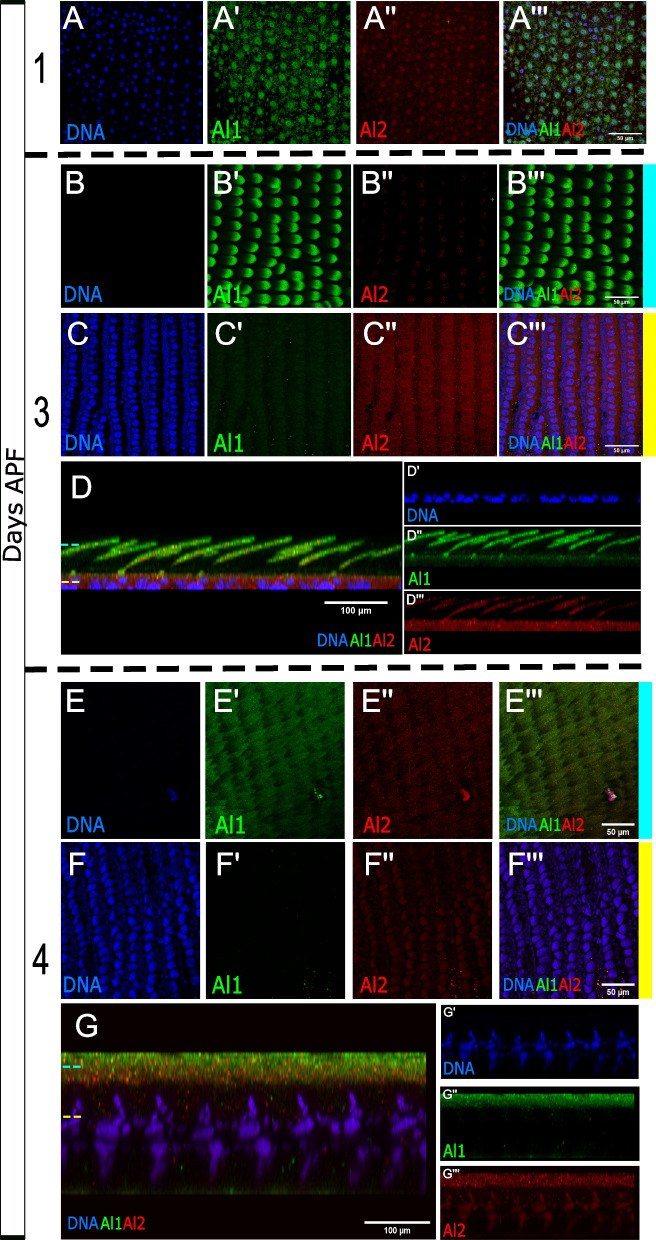
Immunodetection of Al1 and Al2 across pupal development in wild-type *Heliconius cydno*. Immunodetection of Al1 and Al2 is shown in wild-type pupal wings 1 (**A**), 3 (**B–D**), and 4 (**E–G**) days after pupal formation (APF). For 3 days APF a view of the scale (**B**) and nuclei (**C**) z-levels are shown as well as a side reconstruction of the entire scale cell body (**D**). Similarly, for 4 days APF a view of the scale (**E**) and nuclei (**F**) z-levels are shown as well as a side reconstruction of the entire scale cell body (**G**). Panels show detection of DNA (**A**, **B**, **C**, **D′**, **E**, **F**, **G′**), Al1 (**A′**, **B′**, **C′**, **D″**, **E′**, **F′**, **G″**), Al2 (**A″**, **B″**, **C″**, **D″′**, **E″**, **F″**, **G″′**), and a merge (**A″′, B″′**, **C″′**, **D**, **E″′**, **F″′**, **G**). Scale bars are shown on the merged images

We examined Al2 expression during pupal wing scale development (functional cellular units responsible for butterfly wing pigmentation) following previously described detection of Al1 early in pupal wing development [3]. When analyzing pupal scale cells, we observed a trend similar to what we described in embryos. In wings 1 day after pupal formation, we saw strong Al1 expression along the scale buds coming off from the wing blade (Fig. 7A). At this stage, we noticed low levels of Al2 co-localizing with nuclei (Fig. 7A). As development continued, we observed that around 3 days after pupal formation, Al1 was restricted within the developing scale cells extending outside of the wing blade while Al2 was still detected in the nuclei but also appeared around the cells within the wing blade (Fig. 7B–D). Interestingly, Al2 appeared to be excluded from the nuclei of epidermal cells (Fig. 7B–D) which are not believed to be involved in pigmentation [21]. Around 4 days after pupal formation, we observed that Al2 still co-localized with nuclei but it also accumulated on the proximal parts of scale cells (Fig. 7E–G). This was spatially distinct with Al1 which, as development continued, tended to localize more distally within scale cells (Fig. 7E–G). Overall, the detection of Al2 appeared weaker than Al1 across the entire development process of the pupal wing, consistent with our previous measurements of gene expression [27].

## Discussion

**Fig. 8:**
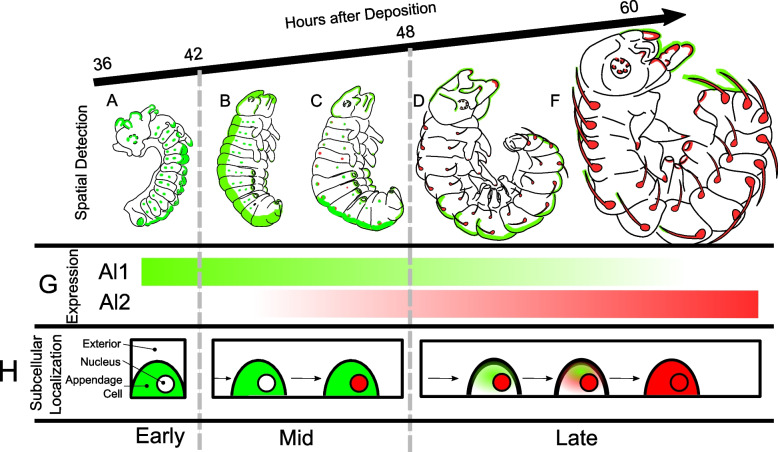
Summary of Al1 and Al2 Immunodetection in *Heliconius cydno* embryos across development. Schematics of the side views of the embryos across development (between 36 and 60 h after egg deposition) are shown. Al1 detection is shown in green while Al2 detection is shown in red within embryos in their early (**A**), mid (**B**, **C**), and late (**D–F**) developmental stages. **G** The bar graphs showcase the temporal shifts in the expression of Al1 and Al2. **H** Schematic representation of the observed subcellular localization for the detection of Al1 and Al2 over time during embryonic development

Our work presents the first characterization of Al2 expression in embryonic appendages, providing a unique point of contrast with the ancestral role of appendage development previously described for *aristaless* in other insects [4, 7, 8, 20, 24] and Al1 in butterflies [3]. We performed this characterization in a comparative framework by further describing the Al1 pattern of expression across multiple stages of embryonic development and including an analysis of Al2 across the same stages (summary in Fig. 8).

Overall, our work describes some similarities between Al1 and Al2 with respect to their association with sensory appendages, possibly indicating similar upstream regulation. Some ancient paralogs, such as *engrailed* and *invected*, are known to be co-regulated during *Drosophila* development through the use of shared *cis-*regulatory elements [9]. Like *en/inv*, *al1/al2* were formed by tandem duplication and remain adjacent in the lepidopteran genome, perhaps suggesting a common mechanism for generating divergent expression patterns between nascent duplicates. However, we also note differences in terms of spatial and temporal control of their expression, perhaps suggesting differences at the functional level when it comes to their role in appendage formation and extension.

Our work characterizes expression differences between Al1 and Al2 within ancestral (sensory appendage formation) and novel (wing pigmentation) roles which, at its core, expands our views related to gene duplication, sub-functionalization, and the evolution of novel gene function [15]. It is well known that gene duplication can often lead to sub-functionalization by alleviating the responsibility imposed on a single copy [10]. This process of sub-functionalization can be associated with shifts in spatial and temporal patterns of expression as a result of divergence in gene regulation between the two duplicates [10, 15]. Following gene duplication and sub-functionalization, it is common to observe that the same overall gene function is maintained but now segmented into different parts of the developing body or across specific time windows as a result of divergent regulation [10]. Neofunctionalization, on the other hand, posits that one copy retains the ancestral function and the other gains a totally new function [10, 15]. Furthermore, additional models exist of an in-between state of these outcomes following gene duplication [15].

Our data showcase an interesting, yet puzzling, case for the study of gene duplication and the evolution of ancestral and novel functions. First, we do not see spatially distinct expression domains in which the copies are restricted to specific appendages. However, we do see a shift in the temporal regulation of Al1 and Al2, suggesting that there has been at least some level of sub-functionalization at the temporal level associated with regulatory differences. However, the puzzling part resides in the strong differences observed in terms of subcellular localization. Al1 does not co-colocalize with nuclei (at least at the analyzed time points) while Al2 is observed to exhibit clear nuclear localization. Given that Aristaless has been described as a homeodomain transcription factor [8, 24], this could suggest that Al2 has maintained the function of transcriptional regulation. Another interesting observation is how the timing of Al2 activation matches the downregulation of Al1. These observations could suggest some antagonistic relationship between the two copies of the gene, as their expression patterns are temporally distinct with little overlap. This antagonistic relationship could provide the basis for novel mechanisms regulating appendage formation.

As we suggested previously [3], it is possible that Al1 is regulating cellular processes outside the nucleus, as seen with other homeodomain proteins like Extradenticle [1]. Furthermore, the entire subcellular dynamics we describe could be even more complex than expected. For example, it has been shown in vertebrates that Aristaless-Related Homeobox (ARX/ALX) genes can exhibit complex subcellular localization because of dimerization (homodimers and heterodimers) events and active sequestration via other transcription factors [11, 25]. Future work should analyze the possibility that Al1 might be involved with the activation or repression of Al2. In both embryos and wings, we observe a progression from Al1 to nuclear Al2, and given that in both embryos and wings knockouts Al1 produces a phenotype, this might imply that extranuclear Al1 activity is important for proper appendage formation and wing color patterning, which could be mediated by Al2. An experiment, where Al1 is knocked out and then Al2 is analyzed, could reveal the regulatory relationship between the two duplicates.

The similarities between the cellular events in embryos and wings might also suggest a similar underlying mechanism for both processes. It is known, for example, that Alx in rodents can cause pigmentation differences by affecting the maturation rate of pigment cells [16]. Along with this idea, we believe similar parallels exist here between Al1 and Al2 regulating appendage growth and their possible role in scale maturation. Scale maturation has been shown to affect pigmentation outcomes via heterochronic shifts [14]. So possible mechanisms involved in appendage growth might also be controlling scale extension and maturation.

Finally, we addressed the main question of whether Al2 is involved in both novel (color patterning) and ancestral (appendage formation/patterning) roles in butterfly development. Our data suggest that both *aristaless* genes play a role in both developmental functions. This is in contrast to the simplest models of neofunctionalization and subfunctionalization, in which paralogs occupy distinct developmental roles. Our results raise an important question about the developmental role of *aristaless* prior to duplication, and whether it was involved in pigmentation at this time. The possibility of this pigmentation role being ancestral has not been properly investigated.

Like our butterflies, multiple vertebrates examples demonstrate a pigmentation role for duplicated *al*-like genes. For instance, in rodents, a specific ALX gene has been shown to control pigmentation outcomes [16]. Meanwhile, in fish, several ALX genes have been shown to specify pigmented cell types [12, 13]. In these systems, like in butterflies, *aristaless* duplications appear to be relevant for the origin/control of color patterning. Furthermore, for ALX in fish, both ancestral functions and novel pigmentation roles appear functional across multiple duplicates [13]. Future work should explore the expression, function, and cellular basis across other related systems with a single *al* or *alx* copy. This could inform us about general principles for why this gene appears to be ideal for pigmentation and color patterning across many systems and how duplication events fit within its functional evolution.

## Conclusions

In summary, our work presents the first characterization of Al2 across butterfly development. Within our comparative framework with Al1, we were able to determine that both copies of the gene appear to be involved with the ancestral role in appendage formation and the novel role in wing color patterning. However, we were able to identify clear differences with respect to the expression timing and the subcellular localization between the duplicates. This suggests a possible level of sub-functionalization. Early embryonic development showed higher expression of Al1. As development progressed, Al1 expression was reduced while Al2 expression increased (Fig. 8). This temporal shift was also accompanied by dynamic cellular accumulation. Al1 was never observed within nuclei while Al2 started accumulating in nuclei and then transitioned to extranuclear subcellular localization (Fig. 8). These expression and subcellular differences were also present in developing wings where Al1 has been shown to control pigmentation outcomes. Our work highlights how unique expression domains and sub-cellular properties relate to the function of Al proteins across embryology and wing patterning. More mechanistic work about possible interactions (between them or with other proteins) and their ability to regulate downstream targets is needed to uncover more information about their function in such apparently distinct roles. More generally, our work provides unique insight into sub-functionalization and mechanisms of gene expression evolution following gene duplication events.

## Methods

### Butterflies rearing

Butterflies were reared in greenhouses at the University of Chicago with a 16 h:8 h light:dark cycle at ~ 27 °C and 60–80% humidity. Adults were fed Bird’s Choice artificial butterfly nectar. Larvae were raised on *Passiflora oerstedii*.

### Embryo fixation and dissection

Eggs were collected from plants between 24 and 36 h after deposition. We adapted the fixation scheme from Brakefield et al. [5, 6]. Eggs were first transferred to 1.5-mL tubes and washed with PBS to remove any dirt. Eggs were then permeabilized and had their chorion removed with 5% Bleach (PBS) for 6 min. Eggs were then washed 5 times for 5 min in PBS to remove the excess bleach. We added 1 mL per tube of a 4% Paraformaldehyde solution (PBS) for fixing for 30 to 60 min. This fixation step was skipped for eggs being used for in situ hybridization and were instead taken into the methanol series directly. Eggs exposed to paraformaldehyde were then washed in PBST (PBS + 0.5% Triton-X100) 2 times for 5 min and then taken into a methanol series (25%, 50%, 75% methanol solutions in PBS at 4 °C). Eggs were then transferred to 100% methanol and stored at − 20 °C for 5 days. Eggs were then transferred using plastic pipettes to a glass dissection plate with pre-chilled 100% methanol for dissection with fine forceps and dissection needles. Dissected embryos were then pipetted carefully into a 16-well tissue culture plate with 1 mL per well of chilled methanol. These embryos were taken back through a 1 mL per well methanol series (75%, 50%, 25% methanol solutions in PBS at 4 °C) for rehydration in the case of antibody staining or maintained in 100% methanol for in situ staining. Then, embryos were washed twice with 1 mL of PBST per well and stored in PBST at 4 °C for antibody staining.

### Butterfly wing dissections

Butterflies were dissected at early pupal stages following Martin et al. [18]. The protocol and adaptations to it were carried out as follows. The pupae were anesthetized in ice for 20 min before dissection. To obtain the pupal wings, the pupae were pinned on the head and most posterior section of the body. The denticle belt was then removed using dissection forceps to allow for easier access to the wing. Then, micro-dissection scissors were used to carefully cut around the wing margin using the pupal cuticle as a guide. The piece of cuticle together with the pupal forewing was removed and placed directly in a 16-well tissue culture plate with 1 mL per well of a 4% paraformaldehyde solution for fixing. Pupal wings were fixed for 30 to 45 min and then cleaned of any peripodial membrane by using fine forceps. After fixation, the tissue was then washed with PBST (PBS + 0.5% Triton-X100) for antibody staining five times, then stored at 4 °C until stained (not more than 30 days).

### *al1* and *al2 *in situ hybridization of *Heliconius embryos*

We designed and synthesized *al1* and *al2-*specific probes using the *H. cydno al1* and *al2* transcript model (selected region shows 100% identity with the target gene transcript model and around 60% identity with the other copy transcript model). 250 base-pair regions from *al1* and *al2* were amplified using primers (al1-forward GTTCCCTCGCAGCCATTCTT; al1-reverse TACGGCACTTCACCAGTTCT; al2-forward CACCTTTAACCCGACCTCCC; al2 reverse GCAGCTCGTGTTCTCTAGCA) by PCR, cloned into a TOPO vector (Invitrogen), and transformed into competent *E. coli* DH5a cells. We grew 3 replicates of 2 positive colonies and extracted DNA using a miniprep DNA extraction kit. We confirmed insert sequences via Sanger sequencing, linearized plasmids using Not1 and Sac1 restriction enzymes (New England Biolabs), and synthesized probes using a reverse transcription kit (Qiagen) with added DIG-labeled nucleotides. The synthesized probes were purified using Qiagen RNAeasy columns.

In situ hybridizations were performed following an adapted version of Ramos and Monteiro’s [23] protocol designed for larval wings. The entire process was carried out in 24-well tissue culture plates. Tissue was stored in cold methanol post dissection and rehydrated into PBT (PBS1X 0.1%Tween20) on the day of the experiment. Tissue was washed 5 times for 5 min with PBT, then incubated in a pre-hybridization buffer (50%formamide, 5XSSC, 0.1% Tween20, and 1 mg/ml Salmon Sperm DNA) for 1 h at 55 °C. 1 mL of hybridization buffer (50%formamide, 0.01 g/ml glycine, 5XSSC, 0.1% Tween20, and 1 mg/mL Salmon Sperm DNA) with approximately 50 ng of the target gene probe peer well and left to incubate at 55 °C for at least 24 h. The tissue was then washed 5 times for 5 min in pre-hybridization buffer and then left washing in pre-hybridization buffer for 24 h at 55 °C. Embryos were then blocked in 1% bovine serum albumin (BSA) in pre-hybridization buffer for 1 h at 4 °C. Anti-DIG antibody was added (1:2000) to each of the wells and incubated overnight at 4 °C. The tissue was then washed with PBT extensively (10 times or more for 5 min) before development with BM-purple (1 mL per well, Roche Diagnostics). Time of development was approximately 20 min at room temperature to 24 h at 4 °C depending on the probe. Stained tissue was imaged using A Zeiss stereomicroscope Discovery.V20 with an AxioCam adapter. Sense probes were used as controls for both duplicates.

### Al1 and Al2 antibody staining of embryos, larval, and pupal wings

We raised polyclonal antibodies against two Al1 peptides and 1 Al2 peptide using the company GenScript (New Jersey, USA). Peptide antigens (Al1-1: QSPASERPPPGSADC, Al1-2: DDSPRTTPELSHA, Al2: CGSGSGMDDEDIPRR) are located in the N-terminal 40 amino acids and share 25% and 30% identity between Al1 and Al2. Polyclonal antibodies were affinity-purified after harvesting and tested for specificity by performing Dot blot tests as described**.**

We performed antibody staining in pupal wings following Martin et al. [18]. We also applied this staining protocol to embryos and made adjustments based on Brakefield et al. [5, 6]. Tissue stored in PBST (PBS, Tritonx) was blocked in 1% BSA in PBST for 2 h, then incubated overnight in 1 mL blocking buffer and Al1- and/or Al2-specific antibodies (1:1000 for pupal wings and 1:3000 for embryos). Tissue was washed twice quickly, then 5 times for 5 min in ~ 0.5 mL PBST, then incubated in 1 mL of the secondary staining solution (goat anti-rabbit-AlexaFluor 488 [Thermofisher] at 1:1000 for Al1 in pupae and 1:3000 for Al1 in embryos, Donkey anti-rat-AlexaFlour 555 [Thermofisher] at 1:1000 for Al2 in pupae and 1:3000 for Al2 in embryos and Hoechst 33,342 at 1:1000 [Thermofisher] in blocking buffer). The tissue was washed extensively and then mounted on glass slides using VectaShield (Vector Labs) on glass slides. Images were collected using a Zeiss LSM 710 Confocal Microscope and processed using Zen 2012 (Zeiss) and ImageJ. For wild-type Al1 and Al2 double antibody staining of embryos, we used and imaged about 5 individuals for all early, mid, and late time points. For wild-type pupal wing stainings, we used forewings from 2 individuals by time point. For Al1 imaging across embryological development, we used a total of 5 embryos across different stages of development between 24 and 36 h after egg deposition.

## Supplementary Information

**Additional file 1. Supplemental Figure 1.** *In situ* hybridization staining against *al1* and *al2* transcripts across *Heliconius* embryonic development. *In situ* hybridization stainings are shown in embryos spanning 36 to 60 hours after egg deposition for both Al1 (A-D) and Al2 (E-H). Multiple embryos of specific stages stained with control sense probes for both genes are also shown (I-L).

## Data Availability

All data generated or analyzed during this study are included in this published article and its supplementary information files. The raw files used and/or analyzed during the current study are also available from EXB and MRK on request.

## Abbreviations

Al: Aristaless
Al1: Aristaless1
Al2: Aristaless2
APF: After pupal formation
A/P: Anterior-posterior
