# Supplementary figures and images for: Divergent expression of aristaless1 and aristaless2 during embryonic appendage and pupal wing development in butterflies
Source: BMC Biol. 2023 May 11;21:104. doi: 10.1186/s12915-023-01602-5 (PMC10173497; doi:10.1186/s12915-023-01602-5)

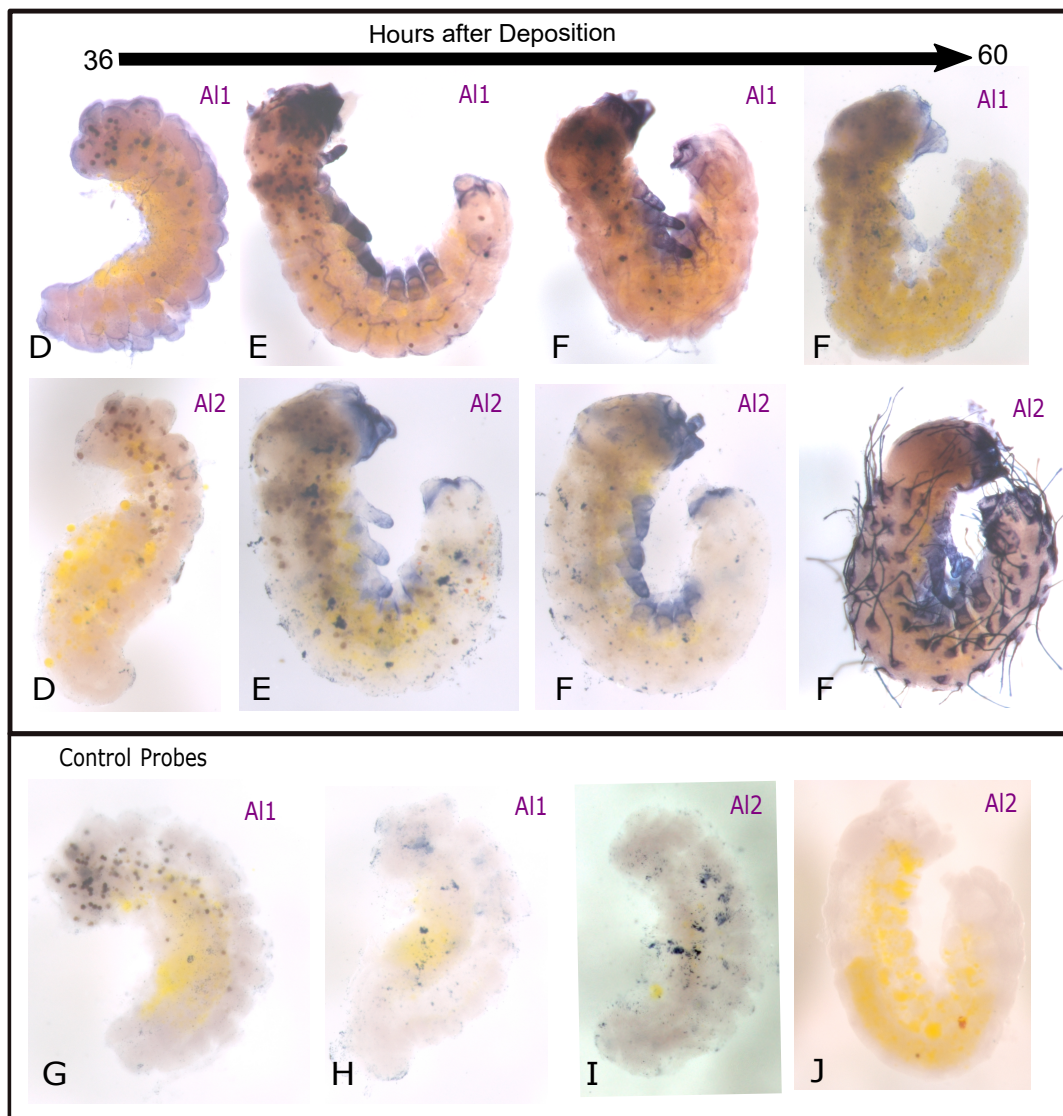

Supplement: Supplementary file 1 — Additional file 1. Supplemental Figure 1. In situ hybridization staining against al1 and al2 transcripts across Heliconius embryonic development. In situ hybridization stainings are shown in embryos spanning 36 to 60 hours after egg deposition for both Al1 (A-D) and Al2 (E-H). Multiple embryos of specific stages stained with control sense probes for both genes are also shown (I-L). [file 12915_2023_1602_MOESM1_ESM.pdf]
